# Needs and preferences of informal caregivers regarding outpatient care for the elderly: a systematic literature review

**DOI:** 10.1186/s12877-019-1068-4

**Published:** 2019-03-13

**Authors:** M. Plöthner, K. Schmidt, L. de Jong, J. Zeidler, K. Damm

**Affiliations:** Center for Health Economics Research Hannover (CHERH), Leibniz University Hannover, Otto-Brenner-Straße 1, 30159 Hannover, Germany

**Keywords:** Informal caregivers, Information, Support, Organization, Preferences

## Abstract

**Background:**

Informal caregivers are an essential pillar for ensuring and maintaining the outpatient care of the frail elderly. Due to demographic changes, including an increase in the number of people in need of care as well as changing social structures (full-time employment of women, increasing number of single households, etc.) these informal care structures are fraught by considerable challenges. To support and facilitate informal caregivers in their role of nursing, it is important to identify their preferences, needs, and thus create a preference-oriented system.

**Methods:**

A systematic review was conducted to identify preferences and needs regarding the organization of informal care. The database searches were performed by using EMBASE, Scopus and Dimdi.

**Results:**

A total of 44 studies were included in the present review. Studies from 17 different countries provide broad international perspectives. Besides the preferences for long-term care structure, the following four principal topics were identified: (1) informational needs; (2) support needs; (3) organizational needs, and (4) needs for societal recognition.

**Conclusion:**

To meet the current challenges in the outpatient or home-based care of elders, it is essential to strengthen the role of informal caregivers. Therefore, it is necessary to adopt and further develop informal care structures according to the needs of informal caregivers. However, demographic, financial and cultural aspects of each country need to be considered as these may influence the preferences and needs of informal caregivers.

## Background

The number of people aged 60 and older is expected to grow from 962 million in 2017 to 21 billion in 2050 [1] in Europe. This global phenomenon affects most countries worldwide, whereas the speed of increase and hence the proportion of elderly differs between countries. The proportion of people aged 65 years and older is expected to grow to an average of 28% in the OECD countries in 2050, while in some countries (e.g. Japan, Spain, Portugal, Greece and Korea) a share of 40% is forecasted. China’s proportion of older people will triple between 2015 and 2050 and also in the USA, Mexico and Israel these growing trends will be influenced by higher rates of fertility and migration. Higher age is associated with higher morbidity, which in turn affects care dependency [2, 3]. Prognosis regarding the number of people in need of care show an increase of 115% in the European Union between 2007 and 2060.The situation in countries outside of Europe is similar. For example the number of people in need of care in the US is expected to double from 13 million in 2000 to 27 million in 2050 [4] and in China, in the worst case scenario, an increase of 115% of individuals in need of long-term care (LTC) is expected between 2015 and 2030 [5]. Generally, elderly care can be organized in inpatient and outpatient care structures. However, in the European Union (EU), 60% of care is provided by informal caregivers [6]. Changing social and structural factors (more individualized household and family structures, increased women’s employment rate, decreased family size, more geographically dispersed families, etc.) will reinforce this situation and will likely lead to a shift from informal to professional care [7]. These trends combined with the growing number of elderly and people in need of care as well as the shortage of formal and qualified caregivers pose a big challenge for the future regarding the structure and organization of long-term care for most countries.

The increasing number of care-dependent people leads to a high economic burden for most healthcare-systems. The principle “outpatient before inpatient” also applies to the nursing of older people in need of care. Institutional care is the primary cost driver in elderly long-term care and costs for long-term care in nursing homes exceed those of home-based care [8]. Hence, enabling care-dependent people to stay in a home-based care setting as long as possible is an efficient cost-cutting strategy for funding agencies.

The majority of the general population wishes to stay at home in old age and would prefer to receive informal care from children or formal care from home assistance services [9]. Informal caregivers are defined as individuals who provide some type of unpaid, ongoing assistance with activities of daily living (ADL), e.g., toileting, feeding, bathing, walking, clothing; or instrumental activities of daily living (IADL), e.g., shopping, meal preparation, housecleaning, and managing finances, for individuals with a chronic illness or disability [10, 11]. However, the decision of family members or relatives to take care of a dependent person, and thus fulfill their wish to age in a domestic environment, are influenced by a variety of factors. The degree of family relationship (children, children-in-law, spouse, etc.) has a significant influence on the willingness of family members to provide care as well as the scope of services [12]. Other aspects that affect the decision to provide outpatient care for a relative are the quality of relationship [13]. (e.g., harmonious or not [14], promises and pacts between the people in need of care and the (potential) caregiver, financial factors, perceptions and attitudes towards nursing homes, health of the caregiver [15], type of impairment of the people in need of care (physical (rates of comorbidity and medical complications) or mental [16], as well as the level of caregivers employment (full-time or part-time job) [17]). In addition, factors such as gender, assistance with elimination, and rearranging work hours affect the use of formal care services by informal caregivers [18]. In the EUROFAMCARE study additional influencing factors for informal care decisions were found. Emotional bonds (e.g., love and affection) (57%), sense of duty (15%) and a personal sense of obligation (13%) were the main reasons for engaging in informal care. Only 3% had taken over the role of caregiving due to the lack of alternatives [19].

Informal caregiving is often associated with negative effects. In the beginning, caregiving is not associated with negative effects [20]. However, several factors lead to an increase in caregivers’ burden, including increased morbidities and higher levels of disability of the care-recipients, hours of care, more variation in caregiving tasks, care setting (care at home vs. institutional care), and gender and age of the caregiver [21]. Beyond demographic or care-specific aspects (type and stage of the disease), organizational aspects (e.g. received support), independence of the caregiver and demands of caring also influence the quality of life of informal caregivers [22]. The estimated nursing time of elderly relatives varies between two and eight years in Germany [23]. It may be a long time with unforeseeable consequences and burdens throughout. Independent of emotional motives such as a sense of responsibility, organizational and structural aspects may influence the decision to provide informal care. Hence, knowing the preferences as well as needs and wishes of informal caregivers enables decision makers to establish care structures meeting the living conditions of those involved and to integrate care into the living environment of the (potential) caregivers.

Facilitations (organizational and structural) in daily processes of caregiving as well as incentives must be established to support individuals’ decision to provide informal care for a care-dependent person. An improvement of the current care arrangements according to the preferences (preferred organization of elderly care) and needs (requirements for organization of long-term care) of informal caregivers can strengthen outpatient care structures. The demographic changes in the society have led to a higher number of elderly and an increase in individuals living in frailty and having greater nursing care needs. This increasing frailty is accompanied by a comprehensive process of adapting to different life situations for both the elderly and their relatives. To meet the demand for the changed service utilization as well as the load limit or breaking point for caregivers, a proactive approach to care planning is necessary [24]. However, to take the load limit and the potential capacities of informal caregivers into account, it is important to understand their motives for providing care to a person in need. Therefore, we conducted a systematic review of literature on informal caregivers’ needs and preferences. Moreover, this review is part of a comprehensive explorative investigation of care preferences and the expected willingness of providing elderly care in the German general population [25].

## Methods

First, we clearly defined the elements of the review (objective/aim, inclusion and exclusion criteria, and outcomes) to focus the scientific issue (Table 1). In December 2016, we conducted a systematic literature search by using the PubMed and Scopus databases as well as the meta-database of the German Institute for Medical Documentation and Information (DIMDI), which constitutes 16 different databases such as Medline, EMBASE, NHS, SciSearch, etc. The search strategy combined English terms for preferences, care, formal and informal care. Additional German terms were used for the systematic literature search in DIMDI. The following search strategy was utilized: [((care* OR geriatric* OR home nursing OR home-dwelling OR old age assistance) AND (elderly OR old* OR aged)) AND relatives OR formal OR informal OR kin OR family*)) AND (OR preferences OR wish OR needs)]. The operator “AND” combined different terms and the truncation “*” was used to achieve a greater coverage.

**Table 1:** Review objective

Objective/ aim	To identify the preferences, needs, and wishes of informal caregivers regarding the organization of care for a care-dependent elderly relative.
Inclusion criteria	Only articles which directly state (explore) the needs/wishes/preferences of informal caregivers regarding the outpatient care of the elderly; articles were not restricted to a special methodology or according to the year of publication.
Exclusion criteria.	Studies focusing on illness-related care (care due to cancer, stroke, etc.; hospital discharge, terminally ill individuals); specific interventions (e.g., prostheses); preferences regarding nutrition, inpatient care, end-of-life care, and palliative care; and post-care studies as well as those focusing only on the quantitative assessment of needs or unmet needs (e.g., in activities of daily living and instrumental activities of daily living) were excluded. Studies focusing on specific types of illness, end-of-life care and palliative care were excluded due to the special care needs for medical and nursing interventions as these are different from elderly care in general.
Outcomes.	Need for information and support, preferences regarding organization of care (e.g., home care or institutional care).

**Fig. 1:**
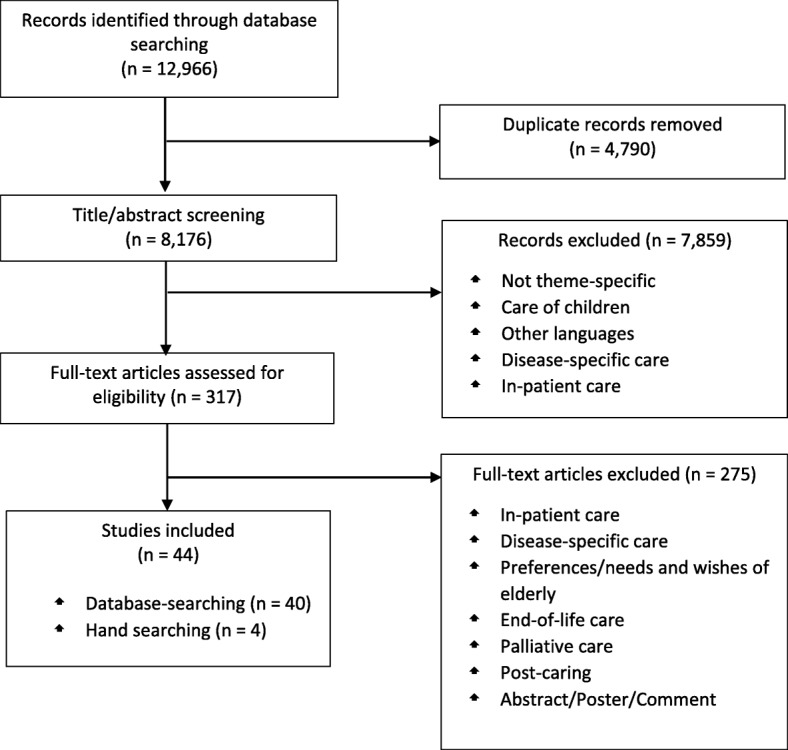
Flow diagram of articles identified and evaluated based on inclusion criteria

An additional search was conducted by hand. According to the PRISMA statement, the assessment was conducted by two independent researchers and disagreements were solved through discussion. Original studies published in full text were included in the assessment. Due to the high number of results, the present systematic review focused on articles pertaining to the perspective of informal caregivers. Results pertaining to care-recipients will be published in a separate overview. Figure 1 summarizes the search process. The contents of the included studies were examined to assign the comprehensive findings to superordinate categories and subcategories.

## Results

The database search using the three databases identified 12,966 records. After removing 4790 duplicates, 8176 titles and abstracts were screened for eligibility, after which, 7859 records were excluded. The remaining 317 records were subsequently assessed for eligibility. Of these, 40 studies that fulfilled the inclusion criteria were included in the final assessment. The hand search resulted in the inclusion of four additional studies (Fig. 1).

### Country, methods, and publication year

A broad international perspective could be achieved by the inclusion of studies conducted in 17 different countries. Thereby, studies from Asia [Malaysia (*n* = 1), Japan (*n* = 2), China (n = 2), Israel (n = 1)], Oceania [Australia (*n* = 4), and New Zealand (n = 1)], Europe [Sweden (*n* = 3), Netherlands (n = 1), UK (n = 3), Ireland (n = 2), Belgium (n = 1), Germany (n = 1), and France (n = 1)], and North America [US (*n* = 12) and Canada (n = 1)] enabled an overview of different cultural perspectives. Two studies conducted an analysis of three countries. Most of the included studies were from the US, whereas two of them analyzed the preferences of caregiving in the Hawaiian context [26, 27].

All included studies were published between 1988 and 2016. Most of the studies appeared in 2014 (*n* = 8), followed by 2016 (*n* = 6), 2012 (n = 6), 2010 (*n* = 5), and 2015 (*n* = 4). Qualitative research (interviews or focus groups) was the most prevalent methodology used.

### Categorization of preferences and needs

The present systematic review identified preferences for the organization and structure of outpatient elderly care as well as various needs of informal caregivers regarding outpatient care within the organization of elderly care. Preferences refer to the organization of care for the elderly and needs to the requirements for the organization of long-term care of informal carers. These findings could be divided into five main topics: (1) preferences for the structure of care, (2) organizational needs, (3) informational needs, (4) support needs and (5) needs for societal recognition. Table 2 provides an overview of the created categories and sub-categories and Table 3 a detailed results overview.

**Table 2:** Overview of the categories and sub-categories created

Preferences	Category	Sub-categories
	Structure of care	Long-Term-Care (LTC)
Needs	Organizational needs	Respite
	Informational needs	Informational needs
		Information sharing
		Professional counselling/advice/educational needs
	Support needs	Need for support
		Support systems
		Robots
	Needs for societal recognition.	Appreciation
		State responsibility

**Table 3:** Detailed Overview of the studies included in the review

Categories	Author (Year)	Country	Aim	Data Collection Methods	Findings
Preferences regarding Long-Term-Care (LTC)					
*Choice of LTC-Arrangements*					
	Wang et al. (2004) [14]	Taiwan	Examination of the preferences of the elderly and their primary family caregivers regarding LTC arrangements.	Questionnaires	Home care is the most preferred LTC arrangement (Home care > institutional care > community based care).
	McCann (1988) [13]	USA	Understanding LTC from the perspective of elderly caregivers, comparison of this perspective with that of nurses and physicians who work with older caregivers and their patients, and learning how nurses and physicians can best address the needs of older caregivers.	Focus groups	Making decisions about home care, the nature of long term home care, and caregivers’ concerns and needs. In general, most spouses want to provide home care for their partners but are often discouraged from doing so by their children and/or by health professionals. Caregivers receive little support in preparation for their roles, and most have little or no contact with the formal health care system.
*Aspects for LTC/ Later life care (LLC) at home*					
	Denson et al. (2013) [28]	Australia	Comparison of the opinions and values of frail elders living at home, younger relatives, and health professionals experienced in discharge-planning, prospectively: Before, not after, an LTC decision.	Interviews (semi-structured and open-ended questions)	Safety/ security; personal (psychological) value of living at home; finances, health; mental and physical abilities; psychological well-being; autonomy, caregiver burden; best interests of the elder; better functioning at home, self-responsibility of the elder, planning.
	Stolee et al. (2014) [29]	Canada	Understanding of views and experiences on later life care (LLC) planning conversations, in terms of (a) respective roles, and (b) barriers and facilitators that should be taken into account when having these conversations.	Interviews (semi-structured)	Effective LLC conversations need to be positive, respectful, and be guided by concerns for the older adult’s well-being.
*Characteristics of Community Directed Care (CDC)*					
	McCaffrey et al. (2015) [78]	Australia	Determining features (attributes) of consumer-directed, home-based support services that are important to older individuals and their informal caregivers to inform the design of a discrete choice experiment (DCE).	Interviews (semi-structured)	The following were Important service characteristics for users: information, choice and control, effective co-ordination and communication, responsiveness and flexibility, and continuity and planning.
Needs within the organization of long term care					
Structural and organizational needs					
Work-life-Balance/ self-care/ Stress management					
	Eldh et al. (2011) [31]	Sweden	Elucidation of the experience of providing informal care to an ageing parent while managing the responsibilities of a working life.	Interviews (narrative)	Providing informal care while working implies seeking a balance between providing support to the parent’s needs and one’s responsibilities at work; possibility for balance by sharing responsibility with others; and the need for frequent and regular dialogue between the managers and the caregivers as employees, on what was currently the most effective arrangements for the workplace and as co-workers.
	Mastel-Smith et al. (2012) [79]	USA	Exploration of caregivers’ educational needs and preferred methods of information delivery.	Focus groups	The need to learn how to balance caregiving and other responsibilities, and care for themselves; respite for time for themselves as a mean of self-care; and the need to be flexible.
	Yedidia et al. (2008) [74]	USA	Elicitation of views of family caregivers regarding expected kinds of assistance from nurses and social workers.	Focus groups	Stress management and coping strategies regarding recognizing and addressing burnout, finding support groups, and accessing a crisis hotline.
Respite					
*Need for respite*					
	Jorgensen et al. (2010) [30]	New Zealand	Reporting the unmet needs.	Telephone interview (scales and open-ended questions)	Need for flexible and reliable respite provision.
	Mackenzie et al. (1996) [32]	China	Gaining personal accounts of experiences of primary caregivers caring for dependent family members.	Interviews (semi-structured)	Short-term respite in an acceptable and appropriate form.
	Feinberg et al. (1999)[50]	USA	Examination of the preferences for and characteristics of consumer-directed (i.e., direct pay) and professionally-managed care (i.e., agency-based) respite for family caregivers of adults with cognitive impairments.	Questionnaires (closed questions and one open-ended question)	Prefer consumer-directed mode (i.e., direct pay) over agency-based in-home respite.
	Eldh et al. (2011) [31]	Sweden	Elucidation of the experience of providing informal care to an ageing parent while managing the responsibilities of a working life.	Interviews (narrative)	Wish for a break from the task of providing support for the ageing parent due to the difficulties of being an informal caregiver and being employed; need for setting one’s own limits.
	Mastel-Smith et al. (2012) [79]	USA	Exploration of caregivers’ educational needs and preferred methods of information delivery.	Focus groups	Need for respite; respite included the need for professional care, family support and other issues; professional caregivers are difficult to find and unreliable.
	McCann (2002) [15]	Ireland	Identification of the views of individuals receiving care (informal caregivers were also interviewed).	Interviews^a^	Need for short-term respite care.
	Lane et al. (2003) [55]	Ireland	Exploration of the perceived health and social care needs of family caregivers of older individuals (including mentally infirm individuals) and exploration of their experience of home care.	Interviews (semi-structured)	Inadequacy of statutory respite services and other services.
*Aspects affecting the acceptance of respite*					
	Greenwood et al. (2012) [33]	UK	Investigation of caregivers’ experiences with, or their perceptions of care workers with respite.	Interviews (semi-structured)	To accept or to use respite trust in the service provider and the individual care worker are very important. The care-recipient must be comfortable and able to communicate well with the care worker; the care worker’s sensitivity to the caregiver’s and care-recipient’s needs and circumstances is an additional important element; they must also be able to respond to any changes in the care-recipient’s condition or mood.
	Lund et al. (2014) [35]	USA	Examining the intervention *Time for Living and Caring (TLC)* in terms of feasibility and potential benefits, and how caregivers viewed their participation.	Survey^a^ including scales and open-ended comments	(1) Weekly or bi-weekly intervention formats were accepted by caregivers. (2) Respite leads to a slight improvement with satisfaction with respite time-use and a slight reduction in burden levels, but no notable changes in satisfaction with caregiving; (3) The participating caregivers recognized the benefits of identifying, in advance, how they wanted to spend their respite time and setting specific goals, which helped empower them to act on their preferences.
	Stirling et al. (2014) [34]	Australia	Assessment of caregiver’s expectations and perceptions of adult day respite services and their commitment to using services.	Interview/survey including rating scales and open-ended questions	Respite decisions depends on the experience and benefits of the care-recipient: (1) want good care; (2) enjoyable experience for the care-recipient; other themes: “want a break.” cost reduction of respite services, and longer opening times for respite services.
Information needs					
Need for information					
	Criel et al. (2014) [36]	Belgium	Identification of the specific needs of the informal caregiver.	Interviews (semi-structured)	Information about existing services
	Jorgensen et al. (2010) [30]	New Zealand	Reporting of the unmet needs	Telephone interview (scales and open-ended questions)	Need for accessible, up-to-date timely information to assess services and support; one national place for accessing information.
	Abu Bakar et al. (2014) [41]	Malaysia	Examination of Malaysian efforts in assisting informal caregivers, based on an analysis of the issues and concerns raised by the caregivers themselves.	Interviews (structured)	Information in solving specific care-giving concerns.
	Hirakawaa et al. (2011) [40]	Japan	Analyzing the priority information needs and sources of family caregivers of home elderly patients.	Questionnaires	Need for information on the public long-term care insurance service (home and institutional care services), and about food and nutrition.
	Mackenzie et al. (1996) [32]	China	Gaining personal accounts of experiences of primary caregivers caring for dependent family members.	Interviews (semi-structured)	Information about rehabilitation and health promoting activities related to emotional, psychological, and physical health; information about sources of community help.
	Zabalegui et al. (2008) [80]	Spain	Better understanding of informal caregivers’ view, particularly about the resources that are available to them, or should be available to them.	Focus groups	Need for information and training (on the process of the illness, the care of the dependent person, and the care of the caregivers themselves, in connection to physical, psychological, and social tasks).
	Zeng et al. (2014) [37]	China	Exploration of the experience of seniors’ family caregivers with regarding the responsibility, burden and support needs during caregiving in Shanghai, China.	Interviews (semi-structured)	Need for information due to the lack of clear information on support services.
	Stockwell-Smith et al. (2010) [38]	Australia	Exploration of the limiting and motivating factors that influence caregivers’ use of respite services and the ability of currently available respite services to meet the needs of caregivers of frail older individuals.	Focus groups	Information need due to the lack of accurate information on community service structures for aged care and formal support services.
	Wilde et al. (2012) [73]	UK	Identification of experiences of home-care reablement service users and their caregivers.	Interviews (semi-structured)	Need for information regarding reablement services (initially and during the reablement).
	Mastel-Smith et al. (2012) [79]	USA	Exploration of caregivers’ educational needs and preferred methods of information delivery.	Focus groups	Need for information on practical aspects of care or caregiving essentials (safety issues regarding the use of mobility aids and other equipment, safe transfer and positioning techniques, and fall prevention); need for disease-specific information; need for information about death and dying, and specifically the prolongation of life and the signs and symptoms of impending death.
	Nickel et al. (2011) [75]	Germany	Exploration of information needs of care-recipients as well as their relatives.	Questionnaire (semi-structured) to document consultation conversations	Need for information on (1) the health care system, (2) individual access options in the health care system, (3) regional service providers, (4) situation and disease-specific aspects.
	Stolee et al. (2014) [29]	Canada	Understanding of views and experiences on LLC planning conversations in terms of (a) respective roles, and (b) barriers and facilitators that should be taken into account when having these conversations.	Interviews (semi-structured)	Desire for information and comfortable mobilizing health care providers in LLC conversations with their care-recipients; information is necessary for making informed choices; useful information types for family members include legal advice, communication strategies, changing roles in their relationship with an older adult, community services and resources, helpful websites, and modifications. These allow their family member to live at home as long as possible.
	Lane et al. (2003) [55]	Ireland	Exploration of the perceived health and social care needs of family caregivers of older individuals (including mentally infirm individuals) and exploration of their experience of home care.	Interviews (semi-structured)	Frustration and hopelessness due to the lack of information
	Yedidia et al. (2008) [74]	USA	Elicitation of views of family caregivers regarding expected kinds of assistance from nurses and social workers.	Focus groups	Information about available services (daytime activities for care recipients, residential facilities, disease-specific services and care coordination); information about drugs.
Information sharing					
	Crotty et al. (2015) [39]	Israel	Identification of how patients older than 75 years and family caregivers of such patients approach sharing of health information, with the hope of applying the results to the development of collaborative patient portals.	Group discussions	Having information would decrease stress; need to acquire information that would help their parent; systems such as patient portals would help assuage some of the stress of caregiving; wish to have access to their elderly parents’ medical records to be able to better coordinate care, appointments, and communication with the family; knowing the activities of the elders; coordinate care for their parents while respecting their preferences and preserving their sense of autonomy.
	LaVela et al. (2016) [81]	USA	Examination and comparison of caregiver perceptions of family centered care by age.	Questionnaire containing closed- and open-ended questions	Want to be informed at different points before, during, and after the patient’s encounters, each representing times at which caregivers need to feel informed and need support; information sharing should be a mutual effort between the caregiver and care provider; younger caregivers require health providers to acknowledge and understand their level of involvement and commitment.
Professional counselling/advices/educational needs					
	Hirakawa et al. (2011) [40]	Japan	Analyzing the priority information needs and sources of family caregivers of home elderly patients.	Questionnaires	Educational need for customer-related issues (problems with customer products and contracts)
	Abu Bakar et al. (2014) [41]	Malaysia	Examination of Malaysian efforts in assisting informal caregivers, based on an analysis of the issues and concerns raised by the caregivers themselves.	Interviews (structured)	Professional counselling and advice from others with similar care-giving-problems
	Fernandes et al. (2013) [27]	Hawaii	Developing and testing a family caregiver training program for Palau in two phases: (1) assessing needs by interviewing key informants and surveying elders and (2) evaluating the caregiver training program that was designed based on findings from the assessment.	Interviews (n/s)	Areas of priority include the need for patient education and training; future training topics include caregiver and family education, wound care, and pain and symptom management
	Van Houtven et al. (2010) [72]	USA	Exploration of the preferences regarding home and community-based services or home-based primary care, (including: quantity and types of tasks provided and desired content for caregiver training programs)	Questionnaires	Interested in participation of caregiver training, especially through (1) phone-based programs (47%) and (2) training at Veterans Affairs.
	Lane et al. (2003) [55]	Ireland	Exploration of the perceived health and social care needs of family caregivers of older individuals (including mentally infirm individuals) and exploration of their experience of home care.	Interviews (semi-structured)	Lack of training for caregivers in relation to lifting and handling skills and a lack of monitoring and support caregivers (in cases of introduction of new or altered medication); the need for clearly systematic caregiver-oriented approaches to tracking, assessment, planning, intervention, and evaluation processes is integral to the strategic development of proactive service plans.
	Yedidia et al. (2008) [74]	USA	Elicitation of views of family caregivers regarding expected kinds of assistance from nurses and social workers.	Focus groups	Learning care tasks (training for bathing and moving, positioning diapers, inserting catheters, using medical equipment, and tailoring care procedures to particular situations); legal advices (negotiating resuscitation preferences and advance directives, and understanding laws applicable to guardianship).
Support needs					
Need for support					
*Type of support*					
	Rodger et al. (2015) [82]	Ireland	Exploration of the experiences of informal caregivers in Ireland and identification the required support in caring for older adults at home.	Interviews (unstructured)	The majority of informal caregiver have inconsistent or no support in caregiving; the following themes emerged: “time is not your own,” duty of care, burden of caring, and support for informal caregivers.
	Lane et al. (2003) [55]	Ireland	Exploration of the perceived health and social care needs of family caregivers of older individuals (including mentally infirm individuals) and exploration of their experience of home care.	Interviews (semi-structured)	Need to support the caregiver’s role.
	Zeng et al. (2014) [37]	China	Exploration of the experience of seniors’ family caregivers with regarding the responsibility, burden, and support needs during caregiving in Shanghai, China.	Interviews (semi-structured)	Caregivers stated that some support services are limited and/or not accessible; there are gaps in caregiver support service; feel sustained mental and emotional confusion and no freedom anymore.
	Stockewell-Smith et al. (2010) [38]	Australia	Exploration of the limiting and motivating factors that influence caregivers’ use of respite services and the ability of currently available respite services to meet the needs of caregivers of frail older individuals.	Focus groups	Selective accepting of assistance; majority of participants accepted the need for assistance but had concerns in terms of trust and quality of support; consistency of formal care services was valued highly.
	Van Dijk et al. (2013) [42]	Netherlands	Exploration of (i) types of informal neighbor support and (ii) experiences of neighbors, volunteers, and professionals providing support.	Interviews (narrative)	Need for professional support for neighbors: providing social monitoring and emotional support; fear of disadvantages and preferred to limit contact.
	Mackenzie et al. (1996) [32]	China	Gaining personal accounts of experiences of primary caregivers caring for dependent family members.	Interviews (semi-structured)	Help in kind (help with tasks on a regular and acceptable basis); adopt of and providing equipment in home for the caring situation.
	Zabalegui et al. (2008) [80]	Spain	Better understanding of informal caregivers’ view, particularly about the resources that are or should be available to them.	Focus groups	Preference of support resources: family group and informal networks are the most helpful and available non-formal resources; want the physician to play a greater role (dedicating more time, having greater tact, etc.), need help for excessive workload and the solitude they suffer as a caregiver; need for physical aids and adaptations; home renovations and help with transport.
	Abu Bakar et al. (2014) [41]	Malaysia	Examination of Malaysian efforts in assisting informal caregivers, based on an analysis of the issues and concerns raised by the caregivers themselves.	Interviews (structured)	Need of help in solving specific care-giving concerns
	Fernandes et al. (2013)[27]	Hawaii	Developing and test a family caregiver training program for Palau in two phases: (1) assessing needs by interviewing key informants and surveying elders and (2) evaluating the caregiver training program that was designed based on findings from the assessment.	Interviews^a^	Access to medication
	McCann et al. (2002) [15]	Ireland	Identification of the views of individuals receiving care (informal caregivers were also interviewed)	Interviews^a^	One-third of the participants expressed the need for more frequent visits from health professionals (e.g., public nurses).
	Browne et al. (2014) [26]	USA	Investigation of health and care preferences that offer the potential for improving well-being in later life for Native Hawaiian elders.	Focus groups	Preferred services: (1) use of community services (when one became familiar through a neighbor or friend); (2) agency personnel (who were culturally informed, professional staff who conducted home visits, services that were affordable and organizations whose policies and procedures were respectful and not intrusive, referred helpers were nurses or social workers, referred respite, family education and support and transportation, more health and prevention programs).
	Yedidia et al. (2008) [74]	USA	Elicitation of views of family caregivers regarding expected kinds of assistance from nurses and social workers.	Focus groups	Communication with professionals (coordination professional help across care sites, collaboration with professionals providing care and finding of compassionate providers); help in recruiting competent help (assistance with checking on qualifications and references and matching available expertise to the needs of the care recipient).
	McCann (1988) [13]	USA	Understanding long term caregiving from the perspective of elderly caregivers, comparison of this perspective with that of nurses and physicians who work with older caregivers and their patients, and learning how nurses and physicians can best address the needs of older caregivers.	Focus groups	Caregivers need more contact with health professionals, and opportunities to share concerns and needs with health professionals.
	Van Kempen et al. (2012) [83]	Netherlands	Exploration of the views and needs of community-dwelling frail older individuals concerning home visits.	Interviews (semi-structured)	Need for home visits of general practitioners; preferences in home visits are the psychosocial context, continuity in professionals, and the patient–professional relationship.
*Emotional/ psychological/social support*					
	Long et al. (2009) [44]	Japan	Comparison of two groups regarding how they became the caregiver, their use of long-term care services and the difficulties, and positive outcomes of caregiving they have experienced.	Interviews	Need for psychological services (counsellors, support groups, etc.)
	Criel et al. (2014) [36]	Belgium	Establishment of a better picture of the various needs of the elderly in their home situation, and a better understanding of the way in which informal care is provided.	Interviews (semi-structured)	Emotional support regarding several problems (emotional, psychological and physically stress as well as practical and organizational problems).
	Milligan et al. (2016) [70]	UK	Gaining a clearer understanding of how (or if) gender plays a part in shaping the forms of formal care support extended to males.	Narrative correspondence: written stories	“Felt a real need and desire to have someone to talk to about the issues, but for this to be delivered through professional services (such as a mental health worker or counselling service).”
	Wailing et al. (1997) [43]	USA	Investigation of whether different dimensions of social support affect mental health via different mechanisms and whether the context in which the support is needed and received will temper its effects.	Interviews (structured questionnaires)	Need for social support in caregiving (to buffer the effects of stress).
	Yedidia et al. (2008) [74]	USA	Elicitation of views of family caregivers regarding expected kinds of assistance from nurses and social workers.	Focus groups	Addressing end-of-life issues, moving the recipient to a facility and dealing with the family.
*Financial assistance*					
	Jorgensen et al. (2010) [30]	New Zealand	Reporting the unmet needs.	Telephone interview (scales and open-ended questions)	Need for appropriate financial support.
	Abu Bakar et al. (2014) [41]	Malaysia	Examination of Malaysian efforts in assisting informal caregivers based on an analysis of the issues and concerns raised by the caregivers themselves.	Interviews (structured)	Financial help with medical costs.
	Mackenzie et al. (1996) [32]	China	Gaining personal accounts of experiences of primary caregivers caring for dependent family members.	Interviews (semi-structured)	Financial help to cover extra costs incurred owing to disability.
	Zabalegui et al. (2008) [80]	Spain	Better understanding of informal caregivers’ view, particularly about the resources that are or should be available to them.	Focus groups	Need for economic support.
	Zeng et al. (2014) [37]	China	Exploration of the experience of seniors’ family caregivers with regarding the responsibility, burden and support needs during caregiving in Shanghai, China.	Interviews (semi-structured)	Caregivers indicate high economic pressure.
	Yedidia et al. (2008) [74]	USA	Elicitation of views of family caregivers regarding expected kinds of assistance from nurses and social workers.	Focus groups	Assistance with financial issues and insurance coverage (locating sources of aid for various income groups, understanding eligibility rules, making health plan decisions, and long-term financing).
Support systems					
*Design/features/requirements of (web-based) support systems/ web-based Apps*					
	Andersson et al. (2017) [84]	Sweden	Exploration of the perceived benefits and challenges with web-based information and communication technologies as a means of supporting working caregivers to fulfill their caregiving role.	In-Depth interviews (semi-structured)	Features of an family-based support network: (1) Support hub for connecting with peers, personnel, and knowledge; emotional support, knowledge bank, and information sources; (2) Experiencing ICT support as relevant in changing life circumstances: links between accessibility, usability and flexibility in support [availability according the caregiver’s life situation (working life and caregiving)]; (3) Upholding one’s personal firewall: utilization is connected to issues of IT security, keeping private matters private in protection of one’s integrity, and ability to feel comfortable in using the system.
	Barbabella et al. (2016) [85]	Italy, Sweden, Germany	Caregivers’ psychological well-being, perceived negative and positive aspects of caregiving, and social support received were assessed before and after the 3-month intervention.	Questionnaires and focus groups	Online social support, role awareness, caregiving activities, psychological well-being, and technical concerns. The analysis suggested that the intervention was useful and appropriate, and it improved self-efficacy and the reappraisal of the caregiver’s role.
*e-health support*					
	Blusi et al. (2014) [45]	Sweden	Investigation of whether caregiver support provided as an e-health service could benefit older family caregivers in rural areas, as compared with conventional non-e-health based support.	Interviews (semi-structured)	Flexibility (choosing suitable information; deciding the time of support, matching support activities with current needs) and availability (always someone to ask, accessible at odd hours, on demand) of the systems are essential to meet the caregivers’ needs.
	Shah et al. (2012) [46]	USA	Documentation of the experiences of patients, their caregivers, healthcare personnel, and staff members with a program that provides telemedicine-enhanced emergency care to older adults residing in senior living communities (SLCs) and to delineate perceived barriers and facilitators.	Interviews (semi-structured)	Telemedicine program eliminates the need to travel to the emergency departments; provision of care immediately.
	Williamson et al. (2014) [86]	USA	Assessing the information and technology needs of Long Distance Caregivers (LDC).	Interviews (semi-structured)	Requirements or design of an App for LDC: Information regarding medication regimens and adherence, calendaring, and cognitive health were most needed. Participants also described needs for video calling, activity data regarding sleep and physical exercise, asynchronous communication, photo sharing, journaling, access to online health resources, real-time monitoring, an overall summary of health, and feedback/suggestions to help them improve as caregivers. In addition, all respondents estimated their usage of a LDC health management website would be at least once per week, with half indicating a desire to access the website from a smartphone.
*In-home-monitoring*					
	Wild et al. (2008) [47]	USA	Identifying of monitoring needs and expectations of community-residing elderly and their family member.	Focus groups	Maintaining independence in the home (identifying and responding to immediate needs (e.g., falls); monitoring to detect cognitive decline over time; sharing information with the physician; trade-off between privacy and usefulness: usefulness regarding safety, maintaining independence and health was valued more as compared to privacy).
Robots					
*Requirements*					
	Bedaf et al. (2016) [57]	Netherlands/ UK/ France	Assessment of the acceptability of robots of elderly individuals.	Focus groups	Autonomy (stay in control of the own life), Agreement (acceptability of a robot), Reminders (medication, motivation for physically activity), Behavior modification (promoting health-promoting behavior), Safety (should keep the user safe), Privacy (robots sharing data with the care team; seen as an extension of the care team), Independence (passive or obedient robot undermine the independence of the user in long term).
*Tasks*					
	Pigini et al. (2012) [87]	Spain	Aiming to generate user requirements and realistic usage scenarios maximizing the alignment with users’ needs, perceptions, feelings and rights of service robots in elderly care at home.	Focus groups and questionnaire	Tasks: monitoring and managing emergency situations, helping with reaching, fetching and carrying objects; using robots in direct physical contact is not appreciated.
	Pino et al. (2015) [88]	France	Investigation of acceptance of socially assistive robots among older adults living in the community.	Questionnaire and focus groups	Services and functionalities were: (a) cognitive support applications to compensate cognitive impairment (e.g., locating lost items, reminding about tasks), (b) communication services to keep an active social life (e.g., video calls, email), (c) risk prevention and healthcare applications (e.g., fall detection, management of critical situations), and (d) applications for supporting everyday tasks (e.g., online grocery shopping, journey planning, simplified Internet access); other functionalities mentioned were entertainment (e.g., music, poetry, and reading) and information and news applications for keeping the user up to date with current events (e.g., broadcast news sources); “life memory albums” available via the robot to support autobiographic memory in persons with memory loss and encourage communication with caregivers and/or family members. Additionally, this application could include multimedia material, such as a genealogical tree, pictures and/or videos of significant moments of the life of the person.
*Design*					
	Pigini et al. (2012) [87]	Spain	Aiming to generate user requirements and realistic usage scenarios maximizing the alignment with users’ needs, perceptions, feelings and rights of service robots in elderly care at home.	Focus groups and questionnaires	Preference of human-like appearance and the possibility of voice-command for controlling the robot.
	Pino et al. (2015) [88]	France	Investigation of acceptance of socially assistive robots among older adults living in the community.	Questionnaire and focus groups	Design: mechanical human-like robot integrating some anthropomorphic facial features within a global mechanical-looking design was preferred.
Needs for societal recognition					
*Recognition*					
	Jorgensen et al. (2010) [30]	New Zealand	Reporting the unmet needs.	Telephone interview (scales and open-ended questions)	Need for recognition for the caregiving role.
	Abu Bakar et al. (2014) [41]	Malaysia	Examination of Malaysian efforts in assisting informal caregivers, based on an analysis of the issues and concerns raised by the caregivers themselves.	Interviews (structured)	Recognition and respect, support and encouragement, appreciation and understanding.
	Mackenzie et al. (1996) [32]	China	Gaining personal accounts of experiences of primary caregivers caring for dependent family members.	Interviews (semi-structured)	Acknowledgement of the importance of the caregiver’s job.
	Eldh et al. (2011) [31]	Sweden	Elucidation of the experience of providing informal care to an ageing parent while managing the responsibilities of a working life.	Interviews (narrative)	Society should acknowledging caregivers better for providing support and care for their ageing parents.
State responsibility					
	Browne et al. (2014) [26]	USA	Investigation of health and care preferences that offer the potential for improving well-being in later life for Native Hawaiian elders.	Focus groups	Caregiving as a shared responsibility of the family and government; the government should have a role in taking care of elders and families (especially in making insurance and medications accessible and affordable); a few said that the government’s care measures goes against the family’s responsibility; receiving help from the family and friends (core Hawaiian value of family and extended networks). Barriers to government care include: (1) real or perceived costs of Services, (2) agency rules and regulations, (3) issues around respect and privacy, and (4) limited specific services.
	Eldh et al. (2011) [31]	Sweden	Elucidation of the experience of providing informal care to an ageing parent while managing the responsibilities of a working life.	Interviews (narrative)	Need for legislation for supporting employees’ rights to provide care; need for such solutions.

^a^: No specific interview type was mentioned

#### Topic 1: Preferences regarding the structure of elderly care

Preferences for the organization of long-term care (LTC) was a recurring subject of interest in the literature. Regarding the choice of LTC-arrangements, Wang (2004) found that home care is the most preferred arrangement for caregivers as well as care-recipients. Home care, institutional care, and community-based care represent—in this order—the preferred organization of nursing. A study by McCann (1988) rated home-based care as the preferred caring option for spouses. Aspects for LTC or later life care (LLC) were evaluated by Denson et al. (2013) and Stolee et al. (2014). Security, personal (psychological) value of living at home, finances, health, mental and physical abilities, psychological well-being, autonomy, caregiver burden, best interests of the elder, self-responsibility of the elder, and planning were some important factors that influenced caregivers’ LTC decisions [28]. Stolee et al. (2014) identified the need for positive and respectful LLC conversations and for care decisions to be guided by concerns for the care-recipient’s well-being [29].

#### Topic 2: Organizational needs

In the category of organizational needs, topics such as work-life-balance and respite were often mentioned. Providing informal care while being employed requires a balance between the needs of the care-dependent person and the workplace responsibilities of the caregiver. Eldh et al. (2011) and Mastel-Smith et al. (2012) found this to be achievable by sharing the care responsibility with others, frequent and regular dialogues between care managers and informal caregivers, and self-care or flexibility. The need for respite (time for self-care) [30] and a wish for a break [31] or specifically for short-time respite [15, 32] were identified. Mastel-Smith et al. (2010) defined respite to include, amongst others, professional care, family support, whereas participants stated that professional care was difficult to find. Furthermore, Feinberg et al. (1999) identified a preference for consumer-directed models via direct pay over agency-based in-home respite. Greenwood et al. (2012), Lund et al. (2014), and Stirling et al. (2014) assessed aspects for accepting respite. Trust in the care provider and individual care, the care provider’s sensitivity to the care-recipient and his/her needs, and the care provider’s ability to react to mood and condition changes of the care-dependent were identified as important aspects [33]. Moreover, participants wished for good care and an enjoyable experience for the care-recipient, as well as cost reductions of and longer opening times for respite services [34]. Weekly or bi-weekly visits were preferred intervention formats by caregivers [35].

#### Topic 3: Informational needs

Information was one of the major topics identified in this review. Informational needs, information sharing, and professional counselling/educational needs were defined as sub-categories. A broad range of informational needs were observed, including information about existing services [36] as well as information enabling the assessment of services and support [30]. We found a need for care-related information in general [37], for community aged care service structures and formal support services [38] in the included literature. Jorgensen et al. (2010) recommended the implementation of one national place for information in New Zealand. Wilde et al. (2012) analyzed informational needs regarding reablement services. Mastel-Smith et al. (2012) found specific informational needs concerning caregiving essentials (safety issues in terms of mobility and fall prevention, etc.), information about diseases, death and dying. In the subcategory information sharing, themes such as wishes for medical information of the care-dependent parent for the coordination of care [39] and information sharing as a mutual effort between caregiver and provider were discussed. As a third aspect, caregiver counselling or education was recommended according to customer-related issues [40], as well as medical issues like wound care and symptom management [27]. Abu Bakar et al. (2014) identified a need for professional counselling and advice from others with similar problems in caregiving, while Van Houtven et al. (2010), who assessed different types of training for caregivers of frail US veterans, found that participants preferred phone-based training programs over training at the Veterans Affairs.

#### Topic 4: Support needs

Support emerged as a second major topic in connection to the organization of elderly care. Studies included here analyzed the need for support in general as well as for special types of support (such as social or financial assistance), special systems (like e-health), or support by robots.

The general need for support was assessed, for example, by Rodgers et al. (2015), Lane et al. (2004), and Zeng et al. (2014). Stockwell-Smith et al. (2010) identified concerns in terms of trust and quality of support regarding the acceptance of care support. Help in solving caregiving-related problems [41], help to access medication [27], as well as the desire for more frequent visits from health professionals [15] could be identified as specific wishes of informal caregivers. An evaluation of neighborhood caregiving, which is composed of social monitoring and emotional support, showed fears in terms of disadvantages and the preference to limit contact with the care-dependent neighbor [42]. Zabalegui et al. (2014) asked informal caregivers about their preferences regarding support resources. Family groups and networks were seen as the most helpful and available non-formal resource. Types of support mentioned in the focus groups included help for transport, home renovations, need for excessive workload, physical aids and adaptations, among others. Emotional/psychological and social support was classified as another subcategory in terms of support. In the study by Criel et al. (2014) the wish for emotional support due to emotional, psychological, and physical stress, as well as practical and organizational problems was stated. Furthermore, social support in caregiving to buffer the effects of stress [43] and the need for psychological services from counsellors and support groups [44] were pointed out.

Studies dealing with preferences for support systems for informal care were also identified. Participants in such studies were interviewed according to the design and requirements of support or web-based support systems. For instance, McCaffrey et al. (2015) analyzed the importance of service characteristics of support systems of Australian users. Information, choice and control, effective co-ordination and communication, responsiveness and flexibility, continuity and planning were mentioned in this context. Andersson et al. (2017) conducted semi-structured in-depth interviews to assess important features of a web-based family support network. A support hub for connecting peers, staff, and knowledge, links between accessibility, usability and flexibility in support as well as a personal firewall according to IT security were pointed out as requirements for the usage. E-health support constitutes a further possibility to facilitate caregivers in their role of caregiving. Blusi et al. (2014), Shah (2012), and Williamson et al. (2014) analyzed the preferences or needs of informal caregivers regarding e-health services, and flexibility and availability were identified as the essential properties of such assistive technologies [45]. Assistive technology services reduce or eliminate the need to travel to emergency departments [46]. Williamson et al. (2014) addressed the requirements and features of APPs for long distance caregivers. Information regarding medication regimens and adherence, calendaring, and cognitive health were stated as most wanted. Furthermore, participants wished for video calling, recording of activity data regarding sleep and physical exercise, asynchronous communication, photo sharing, journaling, access to online health resources, real-time monitoring, and an overall summary of health. A need for feedback or suggestions to improve their role as caregivers was also mentioned. In-home monitoring was named a useful intervention to maintain independence at home. However, the trade-off between privacy and usefulness were discussed, while safety and maintaining independence achieved a higher value than privacy [47].

The usage of robots in the outpatient care of elderly was another topic in the literature. In a study conducted by Bedaf et al. (2016), the requirements of robots in care were discussed in focus groups in the Netherlands, UK, and France. Thereby, autonomy (staying in control of one’s own life), agreement (acceptance of a robot), reminders (medication and motivation for physical activity), behavior modification (promote a healthy behavior), safety, privacy (data sharing with the care team) and independence (passive or obedient robots) were identified as important features. Pigini et al. (2012) and Pino et al. (2015) explored caregivers’ acceptance of types of tasks conducted by robots in care. Monitoring and managing emergency situations, assisting in reaching, fetching and carrying objects were preferred or accepted. However, robots with direct physical contact as a possible application area were not appreciated [48]. Furthermore, cognitive support applications (to compensate for cognitive impairment), communication services (to maintain an active social life), risk prevention and healthcare applications (e.g., fall detection), applications to support everyday tasks (e.g., online grocery shopping, journey planning), as well as information sources and applications that enable the user to stay up to date (e.g., broadcast news) and assist them with autobiographic memories, were identified as important functionalities. Additionally, Pigini et al. (2012) and Pino et al. (2015) identified a preference for a human-like appearance of the robots.

#### Topic 5: Needs for societal recognition

Appreciation for the role of a caregiver was a stated desire in four of the included studies (e.g., Jorgensen (2010)), as well as respect, support, encouragement, and understanding [31, 41]. Mackenzie et al. (1996) and Eldh et al. (2011) found the need for acknowledgement of the importance of the caregiver’s job. The responsibility of the state was another subtopic in this category. Participants in the study conducted by Eldh et al. (2010) stated the need for legislation to support employees’ rights to provide care.

## Discussion

In this comprehensive systematic literature review, the needs and preferences of informal caregivers concerning the care of elderly relatives (family, neighbors and friends) were analyzed. We could identify preferences as well as essential needs regarding the organization of long-term care. All these identified needs could be categorized into the following four main categories: organizational needs informational needs, support needs, and needs for societal recognition.

In the current review, home care was the predominant preference regarding the organization of elderly care. Preferences regarding the organization and structure of long-term care for a care-dependent relative are highly dependent on various factors, such as the degree of family relationship (children, children-in-law, spouse etc.) [12]. In most cases, for example, spouses want to care for their partners [14]. However, financial factors, degree of care-dependence, employment etc. may be limiting factors, which obstruct home care and necessitate institutional care or community care [12, 14].

For the organization or design of informal care a few needs could be identified in the systematic review. In particular, factors related to community-based care or the design of long-term-care arrangements, as well as respite, were singled out in the literature. The need for respite services was identified. The need for support has to be clearly differentiated from the need for respite. While the need for support may arise due to dealing with the caregiving situation, such as support in daily care or mental help for caregivers to accept the situation, the need for respite is more complex. Respite serves the caring person for recreation. It constitutes a possibility for primary caregivers to obtain short-time discharge from their caregiving situation (from a few hours up to weeks) at home, at a healthcare facility, or at an adult day center [49]. During that time someone else is taking care of the person in need of care. Caregivers want safety as well as reliable and trustful help for their care-dependent relatives [50]. A few positive effects, e.g. reduction of depression, burden and stress, were associated with using respite interventions [51]. A lack of flexibility (e.g. long waiting times for beds or for respite at home) as well as poor quality were mentioned in studies as reasons for the non-use of respite services [48, 52]. A further barrier for the use of respite services was the lack of trust and confidence in the provider of such services [38]. An intense collaboration and coordination between informal caregivers and formal services may reduce such access barriers. Kaambwa (2015) conducted a discrete-choice experiment (DCE) to evaluate the preferences regarding the organization of community-directed care (CDC) [53]. In a preliminary study conducted by McCaffery et al. (2015), important service characteristics for users (information, choice and control, effective co-ordination and communication, responsiveness and flexibility, and continuity and planning) were identified via face-to-face interviews. These characteristics were used to define the attributes included in the DCE. Here, participants expressed a highly significant preference for individual budget managing, being able to choose some of the workers that provide their day-to-day services, and being able to have fully flexible support workers so that they could receive support for activities such as cleaning, shopping, meal preparation, and gardening [53].

The need for information was commonly found, but it varied from the need for information about existing services to information about sources of community help, to that about rehabilitation. Additionally, the need for information sharing on topics such as medical interventions of the care-recipient and organization of information sharing, as well as educational desires and preferences for professional counselling were identified. The European Charter for Family Carers, an initiative of the European Union, states that access to information and being informed about rights and duties are important for fulfilling the caregiver’s role [19]. Furthermore, in a majority of studies support is a prevalent topic. However, the possibility as well the scope of support are defined by the countries’ regulations and differ, like in China by geographic regions [54]. Hence, basic [55] or specific forms (psychological and emotional support) of support as well as financial assistance may influence the population needs. Only 16% of European family caregivers received or had used services or trainings to improve their skills and knowledge for a better care, whereas 10% of these were satisfied with this training [19]. In some European countries (Netherlands, Belgium, France and Luxembourg) various information sources such as formal information of the health insurances or social care professionals for support services were mentioned, which may influence the use of support services [54]. In addition to transnational differences, the use of formal and informal services can differ, for example from rural to urban environment [56] and across cultures [41]. A majority of studies addressed needs regarding support systems, including web-based ones, e-health-related aspects as well as requirements, tasks, and the design of care robots. Several studies reported the wish of the elderly for home-based care or aging in place [57, 58]. The development of technologies in telecare leads to (new) possibilities of safety and security monitoring, monitoring of health parameters and vital signs, and support by information and communication applications [59]. On one side these technologies, for example ambient-assisted living-technologies (home sensors, alarm systems, etc. [60]), support care-recipients in daily living activities, and, on the other side, support caregivers by enabling them to leave the care-recipient alone at home for a short time. Robots for assisting in health tasks such as bathing and lifting reduce caregiver workload. However, no studies with regard to the usage of robots in home-based care could be identified. However, in 2015, approximately 4700 assistance robots were sold worldwide, and according to forecasts of the International Federation of Robotics in Frankfurt, the sales are expected to increase up to 37,500 by 2019 [61, 62]. This implies an existing demand for such devices.

The recognition of the caregiving role was also identified as a need of informal caregivers in the current review [30, 32, 41]. Recognition was a stated wish, need, or expectation from community members. More specifically, caregiving should be recognized as a time-consuming and stressful situation. The mentioned need for recognition of the caregiving role [30], appreciation, and understanding [32] reflect this. The lack of recognition by the state can lead to less support and cause dissatisfaction with the role of caring, social isolation, self-neglect, and concern for personal safety [63]. Recognition from state for informal caregiving can be derived from policy initiatives or defined benefits for informal care. The types and scopes of state responsibility differ internationally. Differences can be found in financial maintenance obligations, sharing of responsibilities between family and state, support measures, financial support measures (cash-for-care) and in-kind services (e.g. monitoring, home support devices) [64].

Organizational needs regarding LTC depend on the preferred type of care. For example, recognition of caregivers’ burden may lead to an implementation of respite services. However, the use of these respite services depends on the information and trust of the caregivers and may in turn influence work-life balance and satisfaction. In a narrow sense, the work-care balance may influence the caregiver’s work-life balance. Reducing working time or having flexible working hours is often essential to organize the long-term care for a care-dependent relative to equally fulfill the requirements at the workplace. Hence, the caregiver may be able to achieve a well-functioning work-care balance. A clear differentiation has to be made between a functioning work and care-life balance. Respite in this case enables a possibility to rest or to pursue the previous leisure activities.

### Applying findings to the German case

Considering the German national context, about 2.9 million people were in need of care in 2015 according to the German Nursing Insurance Act (SGB XI), 83% of these were aged 65 years and above [65]. These demographic changes will likely lead to an increase in the number of care-dependent people to an estimated 3.4 million by 2030 [66]. The current nursing situation (e.g., staff shortage) is expected to deteriorate leading to a lack of approximately 500,000 caregivers by 2030 [67]. Presently, 73% of the care-dependent people are being cared for at home and in approximately two-thirds of the outpatient care cases, informal caregivers (including family, friends, relatives, and volunteers) take care of the care-dependent individuals alone or in combination with formal care services [68]. Care provided only by informal caregivers is the most common form (1.18 million), followed by caregiving provided only by formal care services (331,616) and a combination of formal and informal care (244,648) [69]. This shows that informal caregiving is an essential pillar in the German setting when it comes to providing care for the elderly, and therefore, it needs to be strengthened to tackle future challenges.

In recent years, as in many other Western countries, Germany has had a large number of policy initiatives and regulations focusing on the organization of long-term care in the outpatient care sector. Care by informal caregivers is with a total of 1.18 million cases in 2014 the most prevalent form of care in Germany [69]. Based on the socio-economic panel (SOEP) (2001–2012), the German Institute for Economic Research (DIW Berlin) found that 5–6% of adults provide informal care and 60% of these are at an employable age. In recent years, the proportion of the working population providing care under 65 years increased from 53 to 66%. This increase was higher in the group of full-time staff than in the group of part-time workers, whereas, generally, full-time employees combine informal care and career less often [69]. Similar to the OECD average, in Germany, unpaid care is often provided for parents (44.2%) and spouses (34.9%), followed by friends (21.5%) and relatives (13.0%). Further, caregivers are predominantly women (OECD, 2011); however, in recent years, an increase of male informal caregivers could be observed [70]. These findings show the relevance of strengthening the informal outpatient care sector.

According to a German statutory provision (SGB XI), the informal caregiver or care-dependent is entitled to several non-cash-benefits (contributions for personal hygiene, mobility, and nutrition), care allowance, services of care and discharge (§45b SGB XI), day and night care, short-time care, etc. Willemse et al. (2016) found existing support measures to be known and extensively used in Germany. In recent years, German legislation has enacted several laws regarding the organization of informal care to decrease the burden of caregiving and relief informal caregivers. The Long Term Care Strengthening Act was the first step to improve the situation for care-dependent individuals by expanding the insurance benefits in 2015 (The First Long Term Care Strengthening Act (PSG I). The second Long Term Care Strengthening Act (PSG II) focuses on self-reliance of individuals in need of care. An extension of the support in personal and daily care services for patients and a new categorization of care levels, including cognitive limitations as a factor to determine the need for care, was implemented in January 2017. Two further acts, strengthening the informal caregivers’ compatibility of family, care, and profession, were also adopted in 2015. In the Home Care Leave Act (PflegeZG) regulations regarding the working time of employed caring relatives were defined. Employees can be exempted from work or have the opportunity to work part time for a limited time. Furthermore, informal caregivers are entitled to wage replacement. Moreover, major regulation contents of the PSG I and II were significant variations regarding informal care organization, including (1) improved home-care (free care courses and care consulting); (2) time-off (prevention care up to 6 weeks per year); (3) exemption from work (wage-replacement benefits up to ten days, financial compensation for care assistance and reduced working hours up to two years), and (4) better social security (unemployment and age insurance contributions). By means of these regulations the German legislature exercises its duty of care. Regulations to strengthen informal care and to compensate for the negative effects of caregiving meet the German conditions of the social security statute book to provide social justice and to avert or compensate particular burdens of life (§1 SGB I). Adjustments to informal care conditions contribute equally to the fulfillment of the aim of the Tallinn-Charta of the World Health Organization (WHO) stating that health systems have a duty to maintain health and prosperity [71].

Some of these political national measures initiated in recent years addressed the identified preferences and needs of informal caregivers. LTC at home was the most preferred type of care identified in the review, which is also reflected by the high number of informal caregivers in the current care setting. Therefore, a large number of support services were implemented in 2015 to disburden informal caregivers. Needs regarding the organization of LTC, in terms of work-life-balance or respite, were essential aspects for nursing relatives. The possibility of respite and reducing working time may influence self-care, stress and work-life-balance of informal caregivers. However, needs for accepting respite such as trust and satisfaction are difficult to achieve with legal guidelines. Trust in formal caregivers, for example, is very subjective and can therefore heavily influence the acceptance of respite. The need for information was also addressed by the FfZG in 2015. For instance, in an effort to provide information regarding solving specific caregiving concerns [72], reablement services [73], available services [74] or regional service providers [75], each care-dependent’s health insurance covers the costs of care consulting and caregiving courses. Educational and professional counselling needs, such as learning care tasks (e.g. training for bathing and moving [74] or handling skills [Lane] were also covered by the implementation of care consulting. The need for support in general or specifically emotional, psychological and social support may also be an area of responsibility for professional counselling. Such consultations can be a central part of the training as well as the placement of other contact points such as self-help groups [Long]. Financial assistance were an additional need identified in the systematic review. In Germany, due to the introduction of a new classification system for the severity of care dependency, a wider access to financial support, e.g. for people with dementia, were created as the classification system not only assesses physical limitations in activities of daily living, but also cognitive impairments. However, with respect to support systems such as robots or in-home-monitoring, no concrete measures were defined by the legislation. Nonetheless, the scope of reimbursement of costs for support systems is made on a case-by-case decision depending on the healthcare insurance and the classified severity of care dependency. From the changes in recent years, the growing political will of the state to strengthen informal care can be deduced. However, needs of informal caregivers may differ by region (urban or rural) based on aspects such as availability and accessibility of formal home care services or inpatient LTC-arrangements.

Care, as a meaningful act in the context of life and family history [76], is a very sensitive and complex process demanding a lot from the ones involved, particularly family caregivers. Socio-demographic developments justify the demand for structures and services that allow for a combination of care and support activities, employment and private life, as well as the integration of familiar structures, including external familiar structures such as neighborhood and civil society resources [77].

In general, the preferences regarding structure and organization of long-term care as well as the needs for informal care vary from case to case. Germany is presented here as a case example. Of course, the relevance of the preferences and needs identified in this review also depend on country-specific circumstances. These include demographic aspects (e.g. average age of the population), financial and cultural aspects (e.g. care as family responsibility) as well as the current form of care in the country. In order to get a reliable picture of preferences and needs, each country or even region has to be considered individually. The previously mentioned influencing factors should, however, be taken into account when determining the needs and preferences of certain populations.

### Limitations

The study has several limitations regarding the definition of the inclusion and exclusion criteria. Studies dealing with end-of-life (EOL) care and palliative care were excluded. These settings were assumed to have special care needs for medical and nursing interventions. However, these aspects are essential drivers for need and preferences regarding care. Our study focusses on elderly care in general, whereas EOL care often describes only the care needs at the end of life. The decision to provide care for an individual with a very limited life expectancy may result in other preferences compared to generally elderly care. Hence, also the willingness to provide care may be different. Moreover, some studies lacked a clear distinction between end-of-life care with regard to palliative care and “normal” nursing care for elders. Furthermore, studies with disabled care-recipients were excluded, whereas often a clear distinction of disability in the sense of people in need of care or a previous disability was also lacking. Different needs can be assumed. The included studies were all based on a qualitative survey design. Hence, a correlation between the preferences or needs and the socio-demographic properties of the participants cannot be shown. Caregiving contexts vary, but, due to the limited number of studies as well as their designs, the literature reviews did not enable a comparison between the qualitative studies on types of caregivers (partners vs. children, for example), or working and not-working caregivers, or males and females, or sole caregivers and those sharing caregiving responsibilities. However, further research projects could address these open questions, as they have a significant relevance to the design of informal care provision. Moreover, national regulations and country policies are further aspects, which should be taken into account when interpreting the needs and preferences of informal caregivers. However, due to the low representativeness of some countries in the systematic review, no conclusions can be drawn on the corresponding preferences and needs. These may have a direct impact on organization and structure of informal care and hence, influence preferences and needs of informal caregivers. Furthermore, the defined search strategy led to a large number of results, requiring the separate publication of the perspectives of informal caregivers and care-dependents.

## Conclusion

The proportion of the elderly in the general population is increasing steadily in Germany and worldwide. The growing demand for nursing care and the lack of qualified caregivers requires further strengthening of outpatient care structures. Establishing an elderly outpatient care system, which supports families and friends in providing elderly care, while meeting the needs and wishes of informal caregivers, is of high relevance. A balanced arrangement of formal and informal care services, combined with an easy and comprehensive access to information, support services, and adequate financial compensations, as well as respect and encouragement for those who undertake informal care, could be an attractive model. Future research should measure the preferences regarding the organization of informal care as well as the usage of formal care services with appropriate techniques.

## Abbreviations

CDC: community-directed care
DCE: discrete-choice experiment
LLC: later-life care
LTC: Long-term-care
US: United States
